# Opuscula Physica et Chemica, pleraque antea seorsim edita, jam ab auctore collecta, revisa, et aucta. Cum Tabulis Æneis

**Published:** 1781-01

**Authors:** 


IV. Torberni Bergman, Chemiæ Profeſſoris et Equitis aurati Reg. Ordinis de Waſa; Acad. Imp. N. C. Regiarumque Academiarum et Societatum, Upſal. Stock. utriuſque, Londin. Goetting. Berol. Gothob. et Lund. Sodalis, Pariſinæ Correſpondentis, *Opuſcula Phyſica et Chemica, pleraque antea ſeorſim edita, jam ab auctore collecta, reviſa, et aucta. Cum Tabulis Æneis.* 8vo. Holmiæ. Tom. I. 404 Pages.

THIS is the firſt volume of a very intereſting work: it conſiſts of eleven diſſertations, which we ſhall review in the order in which they are printed. The firſt of theſe eſſays treats *de acido aereo*, or as it is commonly, though improperly called, *fixed air*. In this difcourfe, amidft a great variety of matter with which, as might be expected, the chemical reader is already well acquainted, we meet with many new and ingenious obfervations; we fhall content ourfelves with felecting from it fuch as are not generally known. With regard to the name of *acidum aereum*, which he has given to this fluid, our author remarks that it feemed to him to be the moft proper of any, as there are different kinds of fixed air, and the term *mephitic air* is applicable to any kind of air unfit for refpiration. In order to procure a pure acidum aereum, he recommends it to be twice paffed through water, that it may be thoroughly deprived of any heterogeneous acid combined with it. He obferves, that whatever acid we employ, the refult will be the fame, provided no *acidum fumans* is made ufe of, fo that if the nitrous or marine acids are had recourfe to, we muft be careful to dilute them fufficiently. For the extrication of his aerial acid, our author prefers the pellucid tranfparent calcareous fpar to chalk, the latter being almoft always contaminated with marine acid. When this procefs is carried on by means of fire, magnefia, we are told, will be preferable to the calcacalcareous earths, the former yielding the aerial acid much ſooner, and by a gentler heat than the latter. For this purpoſe he uſes a ſmall retort of green glaſs, only an inch in diameter, with a very ſlender neck. He places this veſſel in a crucible, ſurrounded with gypſum. Another method of procuring the aerial acid is by fermentation. For this purpoſe we are directed to take twenty oz. of coarſe ſugar, and the ſame quantity of yeaſt, to which we are to add 200 cubic inches of water in a glaſs veſſel, large enough to contain 350 cubic inches, which is to be placed in a degree of heat equal to 59° of Fahrenheit's thermometer. In ſix or ſeven hours the common air will be expelled from the veſſel. He obſerves, that the acceſs of the external air is not neceſſary for fermentation; it is ſufficient if the air extricated finds an exit.

The aerial acid is always the ſame by whatever method it is procured. When we wiſh to impregnate water with it, our author recommends a heat a little above the freezing point of Fahrenheit's thermometer. In a heat of 41° it takes in ſomewhat more than its own bulk of the aerial acid; in a heat of 50° it abſorbs only a quantity equal to its own volume; and as the heat is increaſed, it takes in ſtill leſs.

The weight of diſtilled water at 36° of Fahrenheit, when compared with water ſaturated with aerial acid, is as 1,0015 to 1,000. This ſaturated water in a heat not much above 32°, is almoſt taſteleſs, but if kept an hour or two in a heat of 59° or 60°, it affords a pungent and agreeable acidulous taſte.

A ſolution of *heliſtropium* in water, diluted ſo as to be of a fine blue colour) a ſtrong ſolution inclining rather to a violet tinge (became evidently red by the addition of $\frac{1}{50}$ part of aerial acid; but the ſyrup of violets, and other blue vegetable juices were not in the leaſt changed by this addition.

The vegetable fixed alkali ſaturated with aerial acid yields quadrangular priſmatic chryſtals, which our author deſcribes as being formed "*apicibus utrimque e duobus triangulis inverſis, tec-*" "*tique inſtar conniventibus, formatis.*" In a temperate heat theſe chryſtals may be diſſolved in four times their quantity of water. They contain 0,32 of water, 0,20 of aerial acid, and 0,48 of alkali. Their taſte is ſlightly alkaline. They precipitate Mercury from ſublimate in the form of a white powder, eſpecially if the powdered chryſtals have for ſome days been expoſed to the open air.

The mineral alkali ſaturated with aerial acid, gives chryſtals of a different form, "*chryſtallos decaedras vel potius octaedras, apicibus duobus oppoſitis, qua maximam partem truncatis.*" They contain 0,16 of aerial acid, 0,64 of water, and 0,20 of alkaline ſalt.

100 parts of pure mineral alkali (deprived of its water and aerial acid) require 77 parts of vitriolic, 135½ of nitrous, and 125 of marine acid for its ſaturation; the proportions with the pureſt vegetable alkali are 78½, 64, 55½.

The chryſtals of volatile alkali ſaturated with aerial acid were leſs regular than the others, but ſeemed to be octagonal. They contain 0,12 of water, 0,45 of aerial acid, and 0,43 of alkali.

The earth, which when ſaturated with vitriolic acid, conſtitutes what we call *ſpathum ponderoſum*, and which our author on that account ſtiles *terra ponderoſa*, we are told, is very different from calcareous earth. Combined with water, it forms a kind of lime-water, an hundred parts of which contain 0,5 of aerial acid, 0,30 of water, and 0,65 of earth. Water diſſolves about $\frac{1}{900}$ part of its own weight of this earth.

Pellucid calcareous ſpar loſes by burning 0,45 of of its weight; 0,34 parts of this are aerial acid, and 0,11 of water: our author for this reaſon conſiders crude calx as a kind of neutral ſalt, and pure calx, or quick-lime, as a real alkaline ſalt. Of this laſt he informs us, that water will diſſolve hardly $\frac{1}{700}$ parts of its weight.

In the ſame manner as vitriolated tartar, gypſum, and other ſalts diſſolve with greater facility in acidulated than pure water, ſo crude calx is found to diſſolve with the greateſt eaſe in water impregnated with the aerial acid.

Speaking of the common magneſia alba, our author obſerves that $\frac{1}{850}$ parts of it diſſolve in water, and that it contains 0,25 parts of aerial acid, 1,30 of water, and 0,45 of pure earth that does not ſeem to diſſolve in water. Water ſaturated with aerial acid diſſolves $\frac{1}{300}$ of its own weight of common magneſia in a heat equal to 59° of Fahrenheit.

Pure *argilla*, or what is commonly called *terra aluminis*, is not diſſolved by aerial acid; but our author obſerves, that this acid ſeems to have ſome effect upon it, when alum is precipitated by alkali ſaturated with aerial acid. This acid has no effect upon ſiliceous earth.

Pure aerial acid, or fixed air, in its elaſtic

ſtate, ftate, does not act upon iron; but our author found that water impregnated with this acid, diffolved $\frac{1}{10500}$ part of the weight of the water, without depriving the iron of its phlogifton. Alkalis perfectly faturated with this acid do not precipitate this iron.

Water faturated with aerial acid not only diffolves zinc, but alfo its calx in great quantity. Alkalis, as above with iron, produce no precipitation.

*Magnefium* (or, as it is perhaps more properly called, *manganefium*, for the fake of diftinguifhing this new difcovered femi-metal from magnefia) is eafily diffolved by water faturated with aerial acid, but its calx is not fo eafily affected. The folution of manganefium afforded a difagreeable fmell, not unlike that of burnt fat.

Of the other metals few will diffolve in this acidulated water, and even thefe few muft be previoufly divided into the moft minute particles by precipitation, otherwife they are not in the leaft affected by it.

From our author's experiments with this acid and inflammable fubftances, it appears that in a heat equal to 50° degrees of Fahrenheit fpirit of wine abforbed twice its bulk of aerial acid, olive oil ſomewhat more than its own bulk, oil of turpentine nearly twice that quantity, and this latter with ſingular avidity. Æther by means of aerial acid, is expanded to twice its uſual bulk, but this expanſion diſappears in water.

The different kinds of hepar ſulphuris, the liquor ſilicum, and the ſoaps, are all of them decompoſed by the aerial acid.

From experiments made with a view to determine the ſimple elective attractions of this acid, the author is enabled to give the following table of affinities:

Terra ponderoſa.

Calx pura.

Alkali fixum vegetabile.

Alkali fixum minerale.

Magneſia.

Alkali volatile.

Zincum.

Manganeſium.

Ferrum.

From all our author's experiments, it evidently appears, that this fixed air, as it has been called, is a real acid, though weaker, and of a nature different from any other. It attracts ſmoke very ſtrongly. He finds that its ſpecific gravity is 6,0006 greater than that of common air, and from this circumſtance he is induced to imagine that this acid abounds more in the lower than in the higher ſtrata of the atmoſphere. This difference he thinks may be owing to the fermentations and other decompoſitions of bodies near the ſurface of the earth, the effect of which is to diſengage more or leſs of this aerial acid. He alſo conjectures that it may be precipitated from the higher regions of the air by lightning.

